# Assessment of Styrene Oxide Neurotoxicity Using *In Vitro* Auditory Cortex Networks

**DOI:** 10.5402/2011/204804

**Published:** 2011-09-07

**Authors:** Kamakshi V. Gopal, Calvin Wu, Ernest J. Moore, Guenter W. Gross

**Affiliations:** ^1^Department of Speech and Hearing Sciences, University of North Texas, P.O. Box 305010, Denton, TX 76203-5010, USA; ^2^Center for Network Neuroscience, University of North Texas, P.O. Box 305010, Denton, TX 76203-5010, USA; ^3^Department of Biological Sciences, University of North Texas, P.O. Box 305010, Denton, TX 76203-5010, USA

## Abstract

Styrene oxide (SO) (C_8_H_8_O), the major metabolite of styrene (C_6_H_5_CH=CH_2_), is widely used in industrial applications. Styrene and SO are neurotoxic and cause damaging effects on the auditory system. However, little is known about their concentration-dependent electrophysiological and morphological effects. We used spontaneously active auditory cortex networks (ACNs) growing on microelectrode arrays (MEA) to characterize neurotoxic effects of SO. Acute application of 0.1 to 3.0 mM SO showed concentration-dependent inhibition of spike activity with no noticeable morphological changes. The spike rate IC_50_ (concentration inducing 50% inhibition) was 511 ± 60 *μ*M (*n* = 10). Subchronic (5 hr) single applications of 0.5 mM SO also showed 50% activity reduction with no overt changes in morphology. The results imply that electrophysiological toxicity precedes cytotoxicity. Five-hour exposures to 2 mM SO revealed neuronal death, irreversible activity loss, and pronounced glial swelling. Paradoxical “protection” by 40 *μ*M bicuculline suggests binding of SO to GABA receptors.

## 1. Introduction

Styrene is a colorless chemical solvent with an aromatic odor. It is extensively used in industries that manufacture polymers, plastics, and resins. Styrene enters the human body through several routes, especially the respiratory system. More than 80% of the inhaled styrene undergoes bioactivation to styrene oxide (SO) by cytochrome P450 mono-oxygenases [1]. SO is widely used also in industries as a diluent for epoxy resins and as a chemical intermediate in the manufacturing of cosmetics, agricultural chemicals, and surface coatings. A review of the literature indicates that there is a considerable amount of evidence that styrene and SO are neurotoxic although the precise mechanisms are unclear and quantitative data are lacking [2]. 

Exposure of low levels of styrene and its metabolites (including SO) may cause irritation of skin, eyes, and mucus membranes, but there is evidence that high doses can lead to neurological disorders [1]. The permissible styrene exposure limit set by the Occupational Safety and Health Administration (OSHA) and the threshold limit value recommended by the American Conference of Industrial Hygienists is 50 ppm (approximately 416 *μ*M) for long-term exposure and 100 ppm (approximately 850 *μ*M) for short-term exposure [3]. There has been no permissible exposure limits set for SO although SO is the most active metabolite formed from styrene. 

Many of the adverse effects of styrene have been attributed to the accumulation of SO [4]. Otoneurologic tests on industrial workers with long-term styrene exposure at levels below 25 ppm (approximately 200 *μ*M) have revealed problems with the vestibular and auditory systems [5]. Other central nervous system problems induced by styrene exposure include vigilance, memory, vision, visuomotor performance, perceptual speed, and central auditory functions [5–7]. Styrene and SO are shown to impede the functioning of various neurotransmitters in the brain including dopamine and serotonin although uncertainties exist about the exact nature of the hindrance caused by these compounds [3, 8, 9]. The cytotoxicity from styrene and SO exposure is thought to be similar to oxidative stress-induced conditions caused by oxidizing protein thiols [4]. Primary cerebellar granule neurons and human neuroblastoma cells exposed to SO (0.3 to 1 mM) induced apoptosis, which can be triggered by oxidative stress [1, 10–12]. There is also evidence that exposure of primary striatal neurons to SO induces synaptic impairments [2], which might be the reflection of morphological alteration of the neuronal cytoskeleton. Furthermore, these data supported the hypothesis of reactive oxygen species initiating the events of SO cytotoxicity. SO is a proven animal carcinogen and is classified as a possible human carcinogen (group 2B) by the International Agency for Research on Cancer [13]. 

Johnson et al., 2006, reviewed nine studies that examined the relationship between occupational exposure to styrene and hearing loss [14]. They found that in seven of the nine studies, there was an association between styrene exposure and hearing loss. Occupational exposure to styrene levels of 40–50 ppm for more than 10 years showed elevated hearing thresholds at frequencies up to 1500 Hz [15]. However, at lower concentrations of styrene (below 20 ppm), no association between exposure and hearing deficit was found. Chen et al., 2008, reported styrene-induced cochlear injury prior to functional loss in an animal model [16]. Exposure to styrene in the presence of industrial noise is shown to have a synergistic effect on the hearing loss incurred by animals and humans [17–19]. Styrene exposure in combination with noise levels within recommended limits has an effect on the auditory system [20]. Chen and Henderson, 2009, have suggested that individual exposure to noise or styrene may cause stress, temporary alteration, or nonlethal injury to cochlear hair cells, but the combination exposure of noise and styrene strengthens the stress on the hair cells, leading to cell death [21]. 

Neurophysiologic testing of brain dysfunction demonstrates reaction time deficiencies in people exposed to styrene [22]. Studies have also shown that styrene has adverse effects on the performance of the central auditory system, including temporal processing skills [14, 23–25]. The European Directive (EU 2003) has specified that risk assessment should include interactions of noise and work-related ototoxic substances such as styrene [26]. NIOSH has recommended establishment of exposure limits for ototoxic chemicals in the presence and absence of noise [27]. Styrene ranks at the top of the list along with toluene as a potentially ototoxic solvent that is widely used in industrial settings [28]. There is, however, a limited understanding of the effects of styrene or its major metabolite SO on the central auditory system. 

This study was undertaken to assess the toxicity of SO using an *in vitro* model of auditory cortex networks (ACNs) growing on multielectrode arrays (MEAs). The objective was to characterize electrophysiological (functional) toxicity and cellular toxicity for acute and subchronic SO exposures and determine if functional toxicity preceded cellular toxicity. Acute neurotoxicity was assessed by serial additions of the SO to mature cultures (21 div or older) that were spontaneously active. The criterion time point selected was 30 minutes at each concentration. For subchronic neurotoxicity assessment, mature cultures that were spontaneously active were exposed to a single concentration of SO for five hours. The concentration-response relationship of SO was compared to exposure levels of styrene seen in industrial settings, since no such levels are currently available for SO exposure.

## 2. Materials and Methods

### 2.1. Cell Culture

The cell culture techniques using ACNs have been published earlier [29–31]. This study was approved by the University of North Texas Institutional Animal Care and Use Committee. Briefly, auditory cortices were dissected from E16-E17 Balb-C/ICR mouse embryos, and were subjected to the standard culturing procedures. The dissociated neurons were seeded at a density of approximately 10,000 cells/mm^2^ on MEAs with substrate-integrated microelectrodes [29]. The networks were maintained in the incubators at 37°C in a 10% CO_2_ atmosphere and fed twice a week using half-medium changes of Dulbecco's modified minimum essential medium (DMEM) supplemented with 5% horse serum. On the day of the experiment, the original medium was completely replaced with serum-free DMEM (stock medium). 

### 2.2. Microelectrode Arrays (MEA)

Previous publications have described in detail the procedures adopted in the in-house MEA fabrication [32–34]. Briefly, the MEAs were photoetched from commercially available indium-tin oxide (ITO) plates (Applied Films Corp., Boulder, Colo, USA) to generate 5 cm^2^ and 1 mm thick plates with 32 amplifier contact strips on either side. The contact strips terminated in the center of the plate in a 0.8 mm × 0.8 mm recording matrix consisting of 64 recording sites (electrodes). The electrode terminals were either arranged in 4 rows and 16 columns or in 8 rows and 8 columns and conductors measured 1000 Å in thickness and 8 *μ*M in width. The processed ITO plates were spin insulated with methyltrimethoxysilane resin and deinsulated at the conductor tips with single laser shots. The exposed metal sites were then gold plated to lower the electrode impedance at 1 kHz to approximately 0.8 mOhms. 

### 2.3. Electrophysiological Recording

The MEA recording techniques used in the study have also been described in detail in previous publications [29, 32]. The matrix region was treated with poly-D-lysine and laminin to support adhesion of dissociated cells. For electrophysiologic recording, only ACNs that were at least three weeks old *in vitro* were used. By three weeks after seeding, the neurons develop shallow, three-dimensional networks with neuronal cell bodies generally situated on top, and neural processes situated above and below the glial carpet [35]. Moreover, neuronal networks grown in this manner remain spontaneously active and pharmacologically responsive for more than 6 months [32]. 

On the day of the experiment, the cultures were maintained at 37 ± 0.5°C on an inverted microscope stage in a special recording chamber [32]. The recording chamber allows for network maintenance in a constant bath of 1 to 2 mL medium and is well suited for rapid medium changes and short-term (24 hours or less) pharmacological studies. The pH was stabilized at 7.4 by passing a stream of humidified 10% CO_2_ in air through a cap fitted with a heated ITO window to prevent condensation, which permitted microscopic observations. Osmolarity was maintained at 300 to 320 mOsm by adding water at a rate of 65 *μ*L/hr via a syringe pump. Neuronal activity was recorded with a two-stage, 64 channel amplification and signal processing system (Plexon Inc., Dallas) with a total system gain set at 10 k. Channels were assigned to 64 digital signal processors with a 40 kHz sampling rate. Waveshapes representing single active units were discriminated via template matching. Up to four different templates per physical channel could be discriminated in real time and assigned to separate logical channels. To follow the behavior of the network before, during, and after test compound application, data were displayed as mean network activity per minute. Channels with best signal-to-noise ratios were selected for further monitoring. 

### 2.4. Experimental Protocol

Following the assembly on the recording chamber, the ACNs were allowed to stabilize in fresh medium consisting of stock DMEM without serum. The spontaneous activity was generally recorded for a minimum of 30 minutes and was termed “reference activity”. Following this period, addition of SO (Sigma-Aldrich, St Louis, Mo, USA) was undertaken, while the ongoing activity was continuously recorded. SO stock solution was prepared in dimethyl sulfoxide (DMSO), since SO is only slightly soluble in water. The maximum DMSO volume in the bath did not exceed 4% of the total bath volume. Control studies were done with DMSO alone to account for its independent effects on the ACNs. For acute experiments, SO dissolved in DMSO was added to the bath with a micropipette to achieve final concentrations ranging from 100 to 3000 *μ*M, mimicking the styrene levels of 12 ppm to 360 ppm in occupational exposures *in vivo* [2]. The network activity was continuously monitored for about 30 minutes prior to the addition of the next dosage to allow for network stabilization. For subchronic experiments, ACNs were exposed to a one-time application of 0.5 or 2 mM SO, and the activity was monitored continuously for five hours before a complete wash (replacement of the medium with fresh stock DMEM) was undertaken. The cells were monitored optically for morphological changes during acute and subchronic experiments. Cell stress was determined by observations of vesiculation, swelling, obscuration of nucleus, and phase brightness. In a subset of experiments, bicuculline (40 *μ*M) was added prior to addition of SO.

### 2.5. Data Analysis

To establish SO-induced changes in the ACNs, mean spike rates were obtained for each concentration level. To quantify the changes induced by SO, the reference activity was compared to network activity at each concentration of SO, and a normalized percent change was obtained. Independent samples *t*-test was used to identify if the reduction in spike activity seen in ACNs exposed to SO was significantly more than the reduction of spike activity in ACNs exposed to DMSO only. A criterion alpha level of 0.05 was adopted for the comparison. All computations were conducted using the Statistical Package for the Social Sciences (SPSS) software. It is important to note that single cells are not reliable indicators of pharmacological responses. Population responses are more fault tolerant and provide representative dose-response functions [36].

## 3. Results

The ACNs used in this study ranged in age from 21 to 42 days *in vitro*, with a mean age of 31 ± 6.6 days. This study evaluated acute (30 minute exposure) and semichronic effects (five hour exposure) of SO on electrophysiological activity and morphological aspects of ACNs. Independent effects of DMSO were first evaluated for reference purposes followed by investigations of the effects of SO dissolved in DMSO. Further, the effects of SO on ACNs exposed to 40 *μ*M bicuculline (a competitive antagonist of GABA_A_ receptors) were also examined.

### 3.1. Acute Effects of DMSO on ACNs

**Figure 1:**
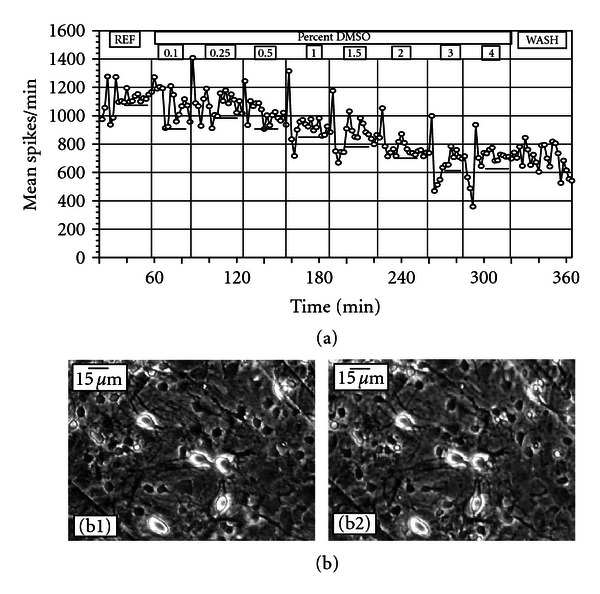
Effects of DMSO on ACN spontaneous activity and morphology. (a) A gradual decrease in mean spikes per min from 26 units. The sudden augmentation of activity seen during the addition of the test compound is due to mixing of the compound in the bath medium. Horizontal bars represent quasistable states used or quantification of activity changes. (b) Neurons and glia in reference medium (b1) and after 200 min in 4% DMSO (b2). No overt changes in neuronal morphology (phase bright cells) can be identified.

Sequential application of DMSO induced a concentration-dependent inhibition of network spike activity.  Figure 1(a) depicts the responses from an ACN that was subjected to DMSO at concentrations ranging from 0.01% to 4% followed by a complete medium change. The average spike rate decreased as a function of DMSO concentration. To identify morphological changes, neurons and neuronal processes in the matrix area were monitored throughout the experiment. No significant morphological changes were identified between reference (no DMSO) and 4% DMSO (Figure 1(b)). 

### 3.2. Acute Effects of SO

**Figure 2:**
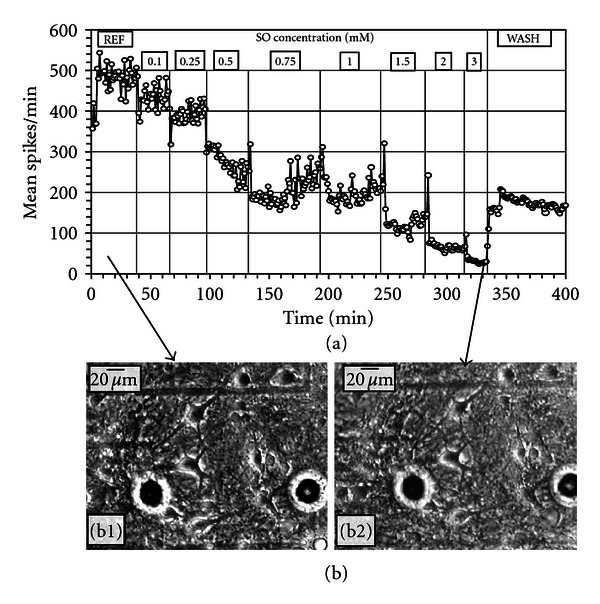
Acute effects of SO on ACN spontaneous activity and morphology. (a) A stepwise dose-dependent inhibition of mean spike rate per min (15 units). The activity was partially reversible with a single wash. (b) Neurons and glia in reference medium (b1) and after 20 min in 3.0 mM SO (b2). No overt changes in morphology can be identified. The round black circles seen in the figures are gold-plated electrodes.

With cumulative application of SO (dissolved in DMSO), a pronounced concentration-dependent spike rate inhibition was noticed.  Figure 2(a) shows a typical ACN response to SO addition: gradual stepwise reduction in average spikes as a function of concentration. At 3.0 mM SO, more than 90% of the spiking activity was lost. When the culture was subjected to a complete wash, there was partial (38%) recovery of the activity. The spike rate IC_50_ (concentration inducing 50% inhibition) occurred at approximately 500 *μ*M. Morphological analysis of the neurons monitored throughout the experiment showed no noticeable changes in cell morphology (Figure 2(b)). 

Table 1 shows the average reduction of mean spike activity in ACNs exposed to DMSO alone (*N* = 3 experiments), and to SO dissolved in DMSO (*N* = 10 experiments). A significantly greater reduction in activity was obtained for ACNs exposed to SO compared to DMSO alone. This difference was significant at the 0.05 level (*t*-test, *P* = 0.03). With a single wash following 3 mM SO application, the activity recovered to only to 28.9 ± 8.9% of the original reference level. This shows that the inhibitory effects of SO remain even after a full medium change.

### 3.3. Concentration-Response Characteristics

**Figure 3:**
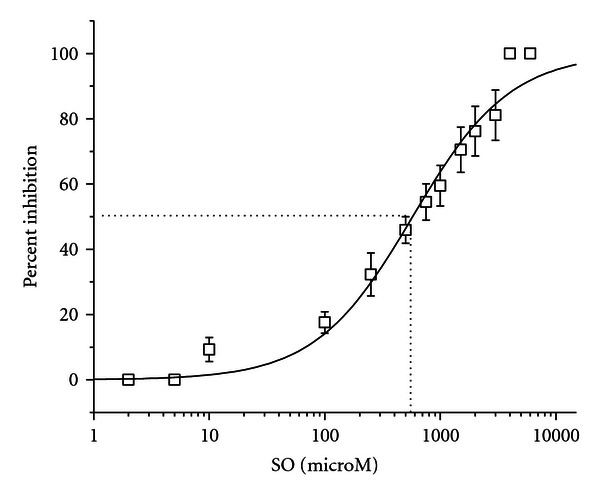
Concentration-response curve for SO. Percent inhibition of network spike rate per concentration of SO was calculated for each network using the specific reference activity of that network and averaged. The spike rate IC_50_ mean ± SE is 511 ± 60.1 *μ*M SO.

The concentration-response curve obtained from 10 ACNs (total of 203 neurons) exposed to acute sequential application of SO is shown in Figure 3. ACNs displayed inhibitory monotonic responses with sequential addition of SO. The IC_50_ value, which represents the mean concentration at which spike rates were inhibited 50% of their original level, was 511 ± 60.1 *μ*M. 

### 3.4. Subchronic (Five-Hour Exposure) Functional and Cellular Effects of SO on ACNs

Since the permissible exposure limit set by OSHA for styrene is 50 ppm (approximately 416 *μ*M), which is close to our IC_50_ concentration of 511 *μ*M, we investigated the effects of a single dose of SO application at a concentration of 0.5 mM on ACNs for a period of five hours (to represent a subchronic condition). Additionally, for comparison purposes, we evaluated the effects of a one-time 2.0 mM sub-chronic exposure. The changes were monitored electrophysiologically as well as morphologically for five hours. 

**Figure 4:**
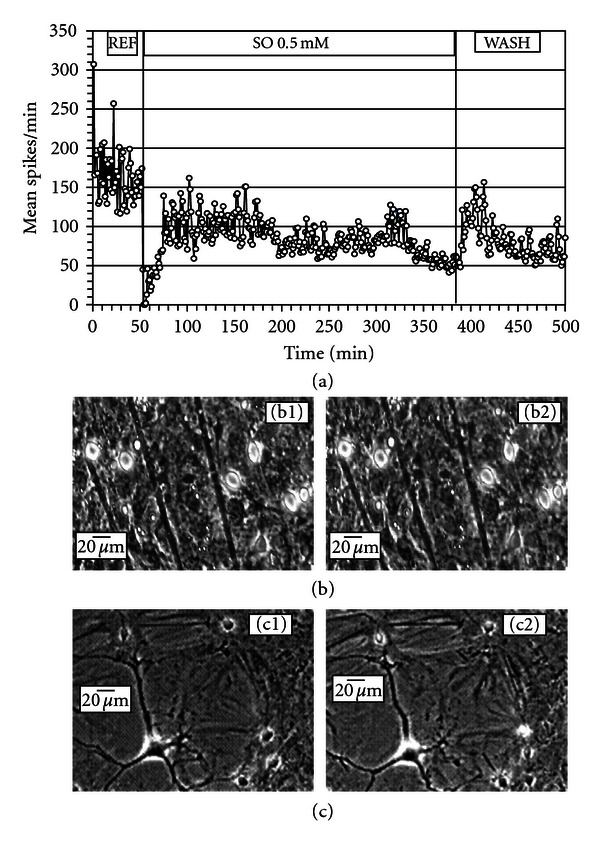
Subchronic effects of 0.5 mM SO (in 0.5% DMSO) on ACN spontaneous activity and morphology. (a) Mean spike rate per min from 33 units show initial cessation of activity followed by recovery of activity that stabilized to about 40% of the original level at the end of five hours. A complete wash did not lead to any further recovery. (b) and (c) Neurons and glia in reference medium (1) and after 5 hrs in 0.5 mM SO (2). Only minor changes in morphology can be identified.

Figure 4(a) depicts the effects of a one-time (nonsequential) application of 0.5 mM SO on an ACN resulting in an immediate cessation of activity. A rapid spontaneous recovery followed, leading to an eventual activity stabilization at approximately 40% of the original reference activity. A complete wash did not facilitate any recovery in this experiment. Morphologically, the neurons appeared to be normal and healthy throughout the duration of the experiment (Figure 4(b)).

**Figure 5:**
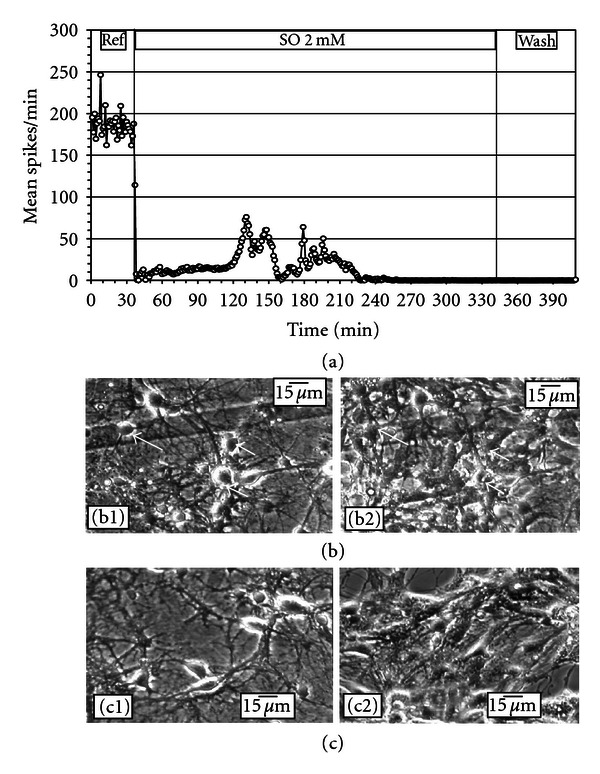
Subchronic effects of 2.0 mM SO (in 2% DMSO) on ACN spontaneous activity and morphology. (a) Mean spike rate per min from 24 units show initial cessation of activity and slow recovery of activity lasting for about an hour that was abolished in 3.5 hrs. A complete wash did not lead to any recovery of activity. (b) and (c) Neurons and glia in reference medium (1) and after 5 hrs in 2.0 mM SO (2). Although some neuronal death is present (arrows) the most obvious response is a massive glial swelling. (b) and (c) Are different networks. c2 is not the same area as c1. Arrows in (b) point to reference neurons for orientation.

Figure 5(a) shows the effect of a one-time (nonsequential) application of 2.0 mM SO on an ACN. The single application of SO caused a more drastic effect compared to the sequential application of SO (as seen in Figure 2(a)). The activity initially ceased, followed by a weak recovery period prior to a complete loss of activity. This was accompanied by a partial loss of neurons and massive glial swelling as seen in Figure 5(b). The glial swelling was so extensive that optical identification of neurons was difficult.

The average spike activity in 3 ACNs exposed to a one-time 0.5 mM for five hours showed reduction to 50.7 ± 8.4% of its original level. Recovery of activity following a complete medium change was less than 50% of the original reference level. Data from 3 ACNs exposed to 2.0 mM SO showed reduction to 92.5 ± 5.8% of its original level within five hours, with no recovery following a complete medium change. 

**Figure 6:**
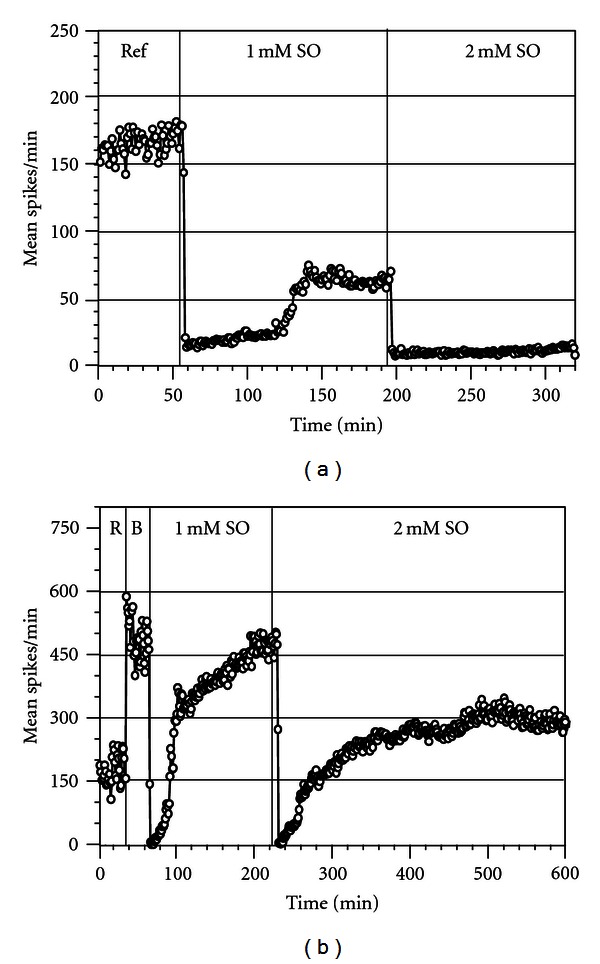
Spontaneous recovery from SO application (1.0 and 2.0 mM) and influence of 40 *μ*M bicuculline. (a) The network shows a 35% recovery at 60 min after 1.0 mM SO application. Note the rapid loss of activity (1-2 min) implying interactions with membrane surface receptors. A 2.0 mM application of SO reduced activity by more than 90%, with no recovery. (b) In the presence of bicuculline, the network shows almost complete recovery to the bicuculline reference with 1.0 mM SO. A 2.0 mM SO application stops all activity followed by a spontaneous recovery to more than 50% of reference. R = Reference; B = Bicuculline.

**Figure 7:**
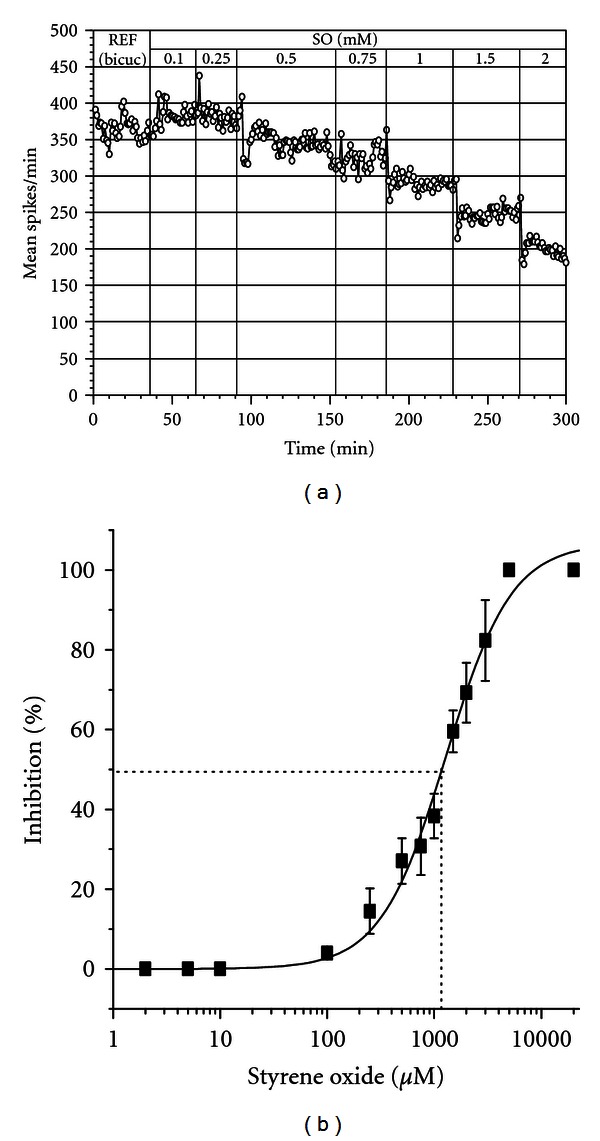
(a) Influence of bicuculline on network response to styrene oxide titration. Reference activity represents pretreatment with 40 *μ*M bicuculline. The concentration-dependent inhibition of spike activity is significantly less in the presence of bicuculline. (b) Concentration response curve from SO titration experiments under bicuculline (*n* = 10). The IC_50_ shifted from 511 *μ*M (Figure 3) to 1405 *μ*M. The slope is close to the standard curve with a slope of 1, implying competitive antagonism at the GABA receptors.

Data obtained so far show a rapid response to the addition of SO that is most obvious at the higher concentrations. The recovery process showed a slow rise of activity of approximately 7% per 10 min and a rapid rise that doubles activity in 10 min (Figure 6(a), 1 mM), suggesting two possible mechanisms. At 2 mM, almost all activity was lost with no recovery. Under 40 *μ*M bicuculline (Figure 6(b)) three different slopes appeared, suggesting three possible different dynamic mechanisms. Further, activity was not lost at 2 mM over an observation period of 350 min. SO titration in the presence of bicuculline (Figure 7(a)) showed retention of about 50% activity at 2.0 mM and even 30% of reference at 4 mM (data not shown). The concentration-response curve (Figure 7(b)) revealed a large shift to higher concentrations with and IC_50_ change from approximately 500 to 1400 *μ*M (*n* = 10 ACNs). The slope of this curve is close to the standard curve (a Hill slope of 1), implying competitive antagonism at the GABA receptor. 

## 4. Discussion

Styrene and its major metabolite SO have been used in industries for more than 100 years. However, their effect on the auditory system has only recently come to the attention of investigators. A strong link between exposure to styrene and “sensorineural” hearing loss, mainly retrocochlear and central problems in industrial workers, has been shown, thus substantiating the notion that styrene is neurotoxic and affects the central auditory nervous system [14, 15, 23–25]. Using multichannel electrophysiological recordings of spontaneously active ACNs growing on MEAs, we tracked the temporal evolution of the effects of SO together with concomitant morphological changes. 

Toxicity of SO was more readily seen using electrophysiological recordings rather than morphological observations. Exposure of ACNs to DMSO induced reduction in electrophysiological activity with no overt signs of morphological damage. The reduction in activity, however, was significantly higher when ACNs were exposed to SO dissolved in DMSO. Addition of SO exhibited steeper concentration-dependent inhibition of network spike activity. Although the IC_50_ value was close to 0.5 mM, morphological analysis of acute exposure to SO up to 3.0 mM concentration did not show any overt signs of morphological damage. This substantiates the fact that electrophysiological recordings provide a more sensitive index of toxic effects than morphological observations. Exposure of 0.5 mM SO subchronically (for five hours) showed approximately a 50% reduction in activity, with no overt signs of cellular damage. However, a complete medium change did not lead to a sizeable recovery, suggesting irreversible electrophysiological effects of SO. Subchronic exposure of 2 mM SO brought about rapid (2 min) cessation of activity and partial neuronal death within five hours. However, it must be noted that when ACNs were exposed acutely to 2.0 mM SO in a sequential (cumulative) manner (Figure 2(a)), the functional effects were not as drastic and the cellular effects were not readily observed compared to the responses seen with a one-time noncumulative application of 2.0 mM SO. The functional changes observed in the concentration-dependent responses cannot be directly attributed to overt neuronal damage, because the morphology of the neurons was not affected at concentration levels that brought about electrophysiological changes. 

An unexpected finding was the extensive swelling of glia (Figure 5) after a five-hour 2.0 mM exposure. The relatively flat glial control carpet erupts into a highly convoluted structure. Quantification of this phenomenon is difficult, as the glia cannot be easily identified in the control state without special staining. However, the swelling represent a major cellular change that is very likely to disrupt cell-electrode coupling. Hence, the loss of activity is at least partially due to this major structural disruption. Although some neuronal death can be morphologically verified, the changes in the glial carpet obscure many details and neuronal death quantification must be performed with staining in a future study. Closer examination also indicates that not all nonneuronal cell types participate. It is possible (but not yet proven) that only astrocytes respond to SO with extensive swelling. It is important to consider that such glial reactions in an animal can lead to a great variety of effects on local blood supply and on electrical activity, culminating eventually in neuronal death.

The almost immediate network response to higher concentrations of SO, the spontaneous recovery, and the “protection” by 40 *μ*M bicuculline (Figure 6(b)) are also surprising but highly repeatable observations. The rapid loss of network activity implies SO interactions with a plasma membrane receptor at synapses. Such a response can be generated by an increase in network inhibition and also by a decrease in network excitation A subsequent spontaneous recovery implies either changes in the pertinent receptor population and/or a compartmentalization of SO, presumably into membranes. The recovery of spontaneous activity following high concentration of SO seemed to include a slow rise of activity followed by a rapid rise (Figure 6(a), 1 mM). At 2.0 mM, most activity was lost with no recovery even after a maximum observed period of 10 hours (data not shown). In the presence of 40 *μ*M bicuculline, three different slopes appeared, suggesting three different dynamic mechanisms (Figure 6(b)), Further, the activity shows retention of about 50% activity at 2.0 mM and even 30% of reference at 4 mM (data not shown). Additionally, the concentration-response curve depicted in Figure 7(b) revealed a major shift of the IC_50_ value in ACNs exposed to bicuculline. The slope of this curve implies competitive antagonism at the GABA receptors. It appears that we are observing the combined effects of (a) SO agonistic activity at GABA receptors, (b) changes in cell/electrode coupling due to glial swelling, and (c) neuronal cell death (at high concentrations). In light of reported reactive oxygen species production by SO [4], it is reasonable to suggest that impaired mitochondrial function and associated impaired ionic gradients lead to osmotic swelling. Such a mechanism should affect all cells but not necessarily at the same rates. 

This study has demonstrated that ACNs exposed to SO show concentration-dependent electrophysiological toxicity even at limits set by OSHA for long- and short-term styrene exposure. Although no overt cellular deficits were noticed for acute or subchronic exposures of 0.5 mM SO, electrophysiological deficits leading to as much as a 50% reduction of activity and incomplete recovery after medium changes signifies the depressant and irreversible effects of SO. However, it is not clear whether impaired reversibility is due to a loss of neurons or to a change in cell-electrode coupling. Styrene exposure, even at concentrations below the OSHA limit, can possibly lead to subtle effects on the auditory system leading to hearing loss and central auditory processing difficulties [5, 37]. Hence, it is recommended that auditory evaluation and monitoring of styrene-exposed individuals should include central auditory test measures. To our knowledge, the swelling of glia has not been reported previously and must be considered a key player in the observed neurological disturbances in humans. Certainly, pressure on neuronal cell bodies or even axons can lead to abnormal evoked activity that could lead to hearing loss and possible associated symptoms such as tinnitus. The glial swelling and the assumed compartmentalization of SO, leading to partial spontaneous recovery, are surprising observations that require further study. 

## Figures and Tables

**Table 1:** Percent average spike rate reduction with acute application of DMSO and SO (maximum DMSO = 3%).

Concentrations		Percent activity decrease		Percent difference
SO (mM)	DMSO (%)	DMSO only (*n* = 3)	SO in DMSO (**n** = 10)	(SO effect)
0.1	0.1	10.8 ± 5.9	17.6 ± 3.3	6.8
0.25	0.25	13.1 ± 8	32.7 ± 6.6	19.6
0.5	0.5	17.1 ± 2.7	45.9 ± 4.1	28.8
1.0	1.0	25.9 ± 6.4	59.5 ± 6.2	33.6
2.0	2.0	36.5 ± 7	76.2 ± 7.6	39.6
3.0	3.0	39.8 ± 8.1	81.1 ± 7.7	41.3
